# 3-[1-(3-Hy­droxy­benz­yl)-1*H*-benzimid­azol-2-yl]phenol dimethyl sulfoxide monosolvate

**DOI:** 10.1107/S1600536812040275

**Published:** 2012-09-29

**Authors:** Magdalena Quezada-Miriel, Alcives Avila-Sorrosa, Juan Manuel German-Acacio, Reyna Reyes-Martínez, David Morales-Morales

**Affiliations:** a Instituto de Química, Universidad Nacional Autónoma de México, Circuito exterior, Ciudad Universitaria, México, DF 04510, Mexico; b Ciencias Básicas e Ingeniería, Recursos de la Tierra, Universidad, Autónoma Metropolitana. Av. Hidalgo Poniente, La Estacón Lerma, Lerma de, Villada, Estado de México, CP52006, Mexico

## Abstract

Crystals of the title compound were obtained as a 1:1 dimethyl sulfoxide solvate, C_20_H_16_N_2_O_2_·C_2_H_6_O. The mol­ecular conformation of the organic mol­ecule is similar to that in the previously reported unsolvated structure [Eltayeb *et al.* (2009 ▶). *Acta Cryst.* E**65**, o1374–o1375]. Thus, the dihedral angles formed by the benzimidazole moiety with the two benzene rings are 57.54 (4) and 76.22 (5)°, and the dihedral angle between the benzene rings is 89.23 (5)°. In the crystal, a three-dimensional network features O—H⋯O, O—H⋯N and O—H⋯S hydrogen bonds, as well as C—H⋯O and C—H⋯π inter­actions.

## Related literature

For potential applications of benzimidazoles in medicine, see: Narasimhan *et al.* (2012 ▶); Alper *et al.* (2003 ▶); Sharma *et al.* (2011 ▶). For coordination compounds of benzimidazole deriv­atives, see: Tellez *et al.* (2008 ▶). For the crystal structure of 3-[1-(3-hy­droxy­benz­yl)-1*H*-benzimidazol-2-yl]phenol, see: Eltayeb *et al.* (2009 ▶).
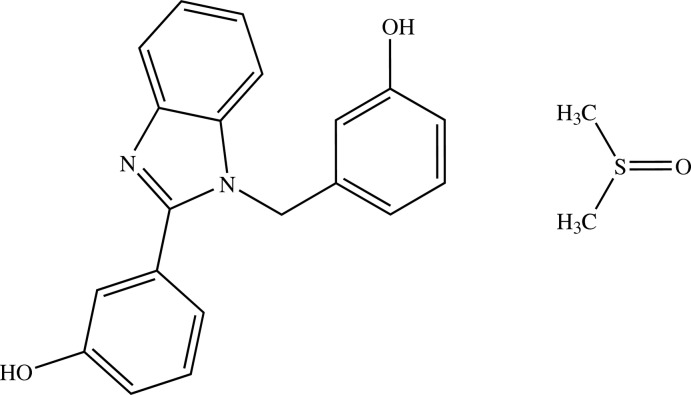


## Experimental

### 

#### Crystal data

C_20_H_16_N_2_O_2_·C_2_H_6_OS
*M* *_r_* = 394.48
Triclinic, 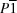

*a* = 8.892 (1) Å
*b* = 9.1951 (10) Å
*c* = 13.1515 (14) Å
α = 85.399 (2)°
β = 71.947 (2)°
γ = 77.442 (2)°
*V* = 997.81 (19) Å^3^
*Z* = 2
Mo *K*α radiation
μ = 0.19 mm^−1^
*T* = 298 K
0.36 × 0.24 × 0.20 mm

#### Data collection

Bruker SMART APEX CCD area-detector diffractometer
Absorption correction: analytical (*SHELXTL*; Sheldrick, 2008 ▶) *T*_min_ = 0.618, *T*_max_ = 0.752
8327 measured reflections
3656 independent reflections
2912 reflections with *I* > 2σ(*I*)
*R*_int_ = 0.046

#### Refinement

*R*[*F* ^2^ > 2σ(*F* ^2^)] = 0.042
*wR*(*F* ^2^) = 0.113
*S* = 1.00
3656 reflections
261 parameters
2 restraints
H atoms treated by a mixture of independent and constrained refinement
Δρ_max_ = 0.23 e Å^−3^
Δρ_min_ = −0.32 e Å^−3^

### 

Data collection: *SMART* (Bruker, 2007 ▶); cell refinement: *SAINT* (Bruker, 2007 ▶); data reduction: *SAINT*; program(s) used to solve structure: *SHELXTL* (Sheldrick, 2008 ▶); program(s) used to refine structure: *SHELXTL*; molecular graphics: *SHELXTL*; software used to prepare material for publication: *SHELXTL*.

## Supplementary Material

Crystal structure: contains datablock(s) I, global. DOI: 10.1107/S1600536812040275/tk5153sup1.cif

Structure factors: contains datablock(s) I. DOI: 10.1107/S1600536812040275/tk5153Isup2.hkl

Supplementary material file. DOI: 10.1107/S1600536812040275/tk5153Isup3.cml

Additional supplementary materials:  crystallographic information; 3D view; checkCIF report

## Figures and Tables

**Table 1:** Hydrogen-bond geometry (Å, °) *Cg*1, *Cg*2 and *Cg*3 are the centroids of the C4–C9, C11–C16 and C17–C22 rings, respectively.

*D*—H⋯*A*	*D*—H	H⋯*A*	*D*⋯*A*	*D*—H⋯*A*
O2—H2⋯O3^i^	0.85 (1)	1.83 (1)	2.6804 (19)	176 (2)
O2—H2⋯S1^i^	0.85 (1)	2.84 (1)	3.6209 (14)	154 (2)
O1—H1⋯N3^ii^	0.86 (1)	1.88 (2)	2.7316 (19)	172 (2)
C18—H18⋯O3^i^	0.93	2.58	3.260 (2)	130
C23—H23*C*⋯O3^iii^	0.96	2.72	3.643 (3)	162
C10—H10*B*⋯*Cg*1^iv^	0.97	2.95	3.622 (2)	127
C5—H5⋯*Cg*2^v^	0.93	2.76	3.624 (2)	156
C23—H23*B*⋯*Cg*3^vi^	0.96	2.86	3.679 (2)	144

Symmetry codes: (i) 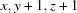; (ii) 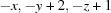; (iii) 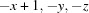; (iv) 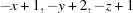; (v) 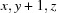; (vi) 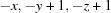.

**Fig. 1.**
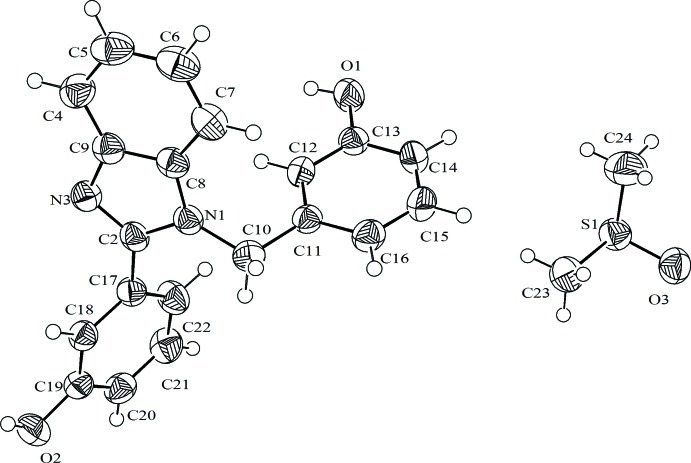
The molecular structure of the title compound, showing 50% probability displacement ellipsoids and the atom numbering scheme.

**Fig. 2.**
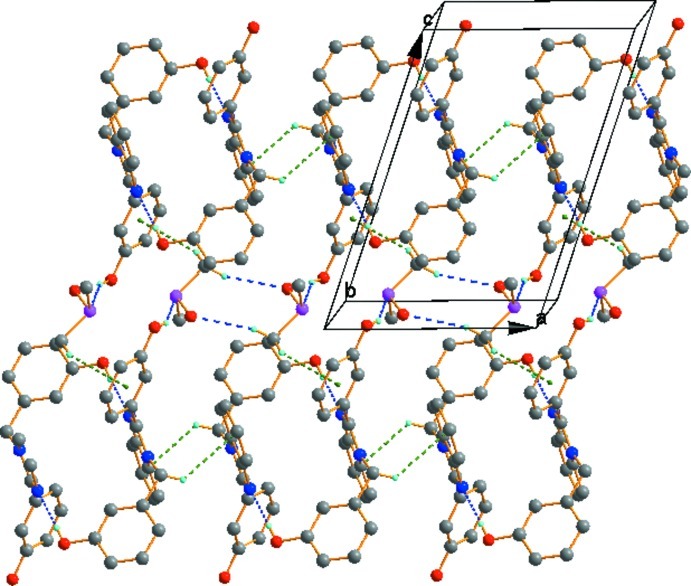
A two-dimensional sheet structure formed through hydrogen bonds interactions parallel to the plane ac, hydrogen bonds are showing by dashed lines.

## supplementary crystallographic information

### Comment

Benzimidazole and its derivatives are of significant importance in medicinal chemistry. In these species, the presence of the benzimidazole heterocycle provides a vast variety of potential biological and clinical applications (Narasimhan *et al.*, 2012). Benzimidazole derivatives with potential biological activities have been widely studied for the treatment of different illnesses such as cancer (Alper *et al*., 2003), infectious diseases, metabolic and cardiovascular disorders, allergies, tuberculosis (Sharma *et al.*, 2011) and different inflammatory conditions. Thus, in order to increase the activity of benzimidazole derivatives its coordination behaviour with transition metals such as Pd(II), Co(II), Ni(II), Cu(II) and Cd(II) (Tellez *et al.*, 2008) has been explored.

The crystal structure of 3-[1-(3-hydroxybenzyl)-1*H*-benzimidazol-2-yl]phenol was first reported by Eltayeb *et al.* (2009). Thus, in this opportunity we would like to report the DMSO solvated structure of 3-[1-(3-hydroxybenzyl)-1*H*-benzimidazol-2-yl]phenol (Fig. 1).

In this molecule, the dihedral angles formed by the benzimidazole moiety with the two benzene rings (C11–C16 and C17–C22) are 57.54 (4) and 76.22 (5)° respectively. The two benzene rings (C11–C16 and C17–C22) are forming a dihedral angle of 89.23 (5)°, these values are similar to those reported previously (Eltayeb *et al.* 2009).

The crystal lattice of the title compound is stabilized by the presence of hydrogen bonds (O—H···N, O—H···O, O—H···S, C—H···O and C—H···π), Table 1. The O—H···N interactions associate two molecules to generate an 18-membered macrocycle with crystallographic inversion symmetry, that are interconnected each other by C—H···π interactions between methylene (C10—H10B) group and the aromatic ring (C4—C9) of the neighbouring molecules, Fig. 2. The C5—H5···π and O—H···O interactions stabilize the three-dimensional arrangement. Finally, the DMSO molecules are associate by C—H···O interactions thus generating eight-membered motifs, by additionally exhibiting interactions with the benzimidazole of the type O2—H2···S1.

### Experimental

To a solution of 3-hydroxybenzaldehyde (0.320 g, 2.0 mmol) in CH_2_Cl_2_, 0.034 g (0.2 mmol) of *p*-toluenesulfonic acid, 0.7 g of *o*-phenylenediamine (6.4 mmol) and molecular sieves were added. The mixture was stirred at room temperature for 24 h. After this time the resulting solution was filtered and the solvent evaporated under vacuum affording 3-[1-(3-Hydroxybenzyl)-1*H*-benzimidazol-2-yl]phenol as a microcrystalline white powder. Single crystals suitable for X-ray diffraction analysis were obtained from a dimethyl sulfoxide solution of the compound.

### Refinement

H atoms were included in calculated positions (C—H = 0.93 Å for aromatic H, C—H =0.97 Å for methylene H, and C—H= 0.96 Å for methyl H), and refined using a riding model, with *U*_iso_(H) = 1.2*U*_eq_ of the carrier atoms. The hydroxyl H atoms were located in a difference map and refined with O–H = 0.85±0.01 Å, and with *U*_iso_(H) = 1.2*U*_eq_(O).

### Crystal data

**Table:**

C_20_H_16_N_2_O_2_·C_2_H_6_OS	*Z* = 2
*M**_r_* = 394.48	*F*(000) = 416
Triclinic, *P*1	*D*_x_ = 1.313 Mg m^−^^3^
Hall symbol: -P 1	Mo *K*α radiation, λ = 0.71073 Å
*a* = 8.892 (1) Å	Cell parameters from 4929 reflections
*b* = 9.1951 (10) Å	θ = 2.5–25.4°
*c* = 13.1515 (14) Å	µ = 0.19 mm^−^^1^
α = 85.399 (2)°	*T* = 298 K
β = 71.947 (2)°	Prism, colourless
γ = 77.442 (2)°	0.36 × 0.24 × 0.20 mm
*V* = 997.81 (19) Å^3^	

### Data collection

**Table:**

Bruker SMART APEX CCD area-detector diffractometer	3656 independent reflections
Radiation source: fine-focus sealed tube	2912 reflections with *I* > 2σ(*I*)
Graphite monochromator	*R*_int_ = 0.046
Detector resolution: 0.83 pixels mm^-1^	θ_max_ = 25.4°, θ_min_ = 1.6°
ω scans	*h* = −10→10
Absorption correction: analytical (*SHELXTL*; Sheldrick, 2008)	*k* = −11→11
*T*_min_ = 0.618, *T*_max_ = 0.752	*l* = −15→15
8327 measured reflections	

### Refinement

**Table:**

Refinement on *F*^2^	Primary atom site location: structure-invariant direct methods
Least-squares matrix: full	Secondary atom site location: difference Fourier map
*R*[*F*^2^ > 2σ(*F*^2^)] = 0.042	Hydrogen site location: inferred from neighbouring sites
*wR*(*F*^2^) = 0.113	H atoms treated by a mixture of independent and constrained refinement
*S* = 1.00	*w* = 1/[σ^2^(*F*_o_^2^) + (0.0674*P*)^2^] where *P* = (*F*_o_^2^ + 2*F*_c_^2^)/3
3656 reflections	(Δ/σ)_max_ < 0.001
261 parameters	Δρ_max_ = 0.23 e Å^−^^3^
2 restraints	Δρ_min_ = −0.32 e Å^−^^3^

### Special details

**Table:**

Geometry. All e.s.d.'s (except the e.s.d. in the dihedral angle between two l.s. planes) are estimated using the full covariance matrix. The cell e.s.d.'s are taken into account individually in the estimation of e.s.d.'s in distances, angles and torsion angles; correlations between e.s.d.'s in cell parameters are only used when they are defined by crystal symmetry. An approximate (isotropic) treatment of cell e.s.d.'s is used for estimating e.s.d.'s involving l.s. planes.
Refinement. Refinement of *F*^2^ against ALL reflections. The weighted *R*-factor *wR* and goodness of fit *S* are based on *F*^2^, conventional *R*-factors *R* are based on *F*, with *F* set to zero for negative *F*^2^. The threshold expression of *F*^2^ > σ(*F*^2^) is used only for calculating *R*-factors(gt) *etc*. and is not relevant to the choice of reflections for refinement. *R*-factors based on *F*^2^ are statistically about twice as large as those based on *F*, and *R*- factors based on ALL data will be even larger.

### Fractional atomic coordinates and isotropic or equivalent isotropic displacement parameters (Å^2^)

**Table:**

	*x*	*y*	*z*	*U*_iso_*/*U*_eq_	
S1	0.19023 (5)	0.03592 (5)	0.01994 (4)	0.05482 (17)	
O1	0.06161 (15)	0.77881 (14)	0.21845 (10)	0.0553 (3)	
H1	−0.002 (2)	0.8399 (19)	0.2668 (13)	0.066*	
O2	0.15779 (16)	0.67344 (14)	0.95005 (9)	0.0580 (3)	
H2	0.201 (2)	0.7438 (18)	0.9583 (16)	0.070*	
O3	0.29883 (16)	−0.10268 (15)	−0.03524 (10)	0.0635 (4)	
N1	0.31522 (15)	0.88121 (14)	0.49775 (10)	0.0394 (3)	
C2	0.21131 (18)	0.90591 (18)	0.59903 (12)	0.0393 (4)	
N3	0.15587 (16)	1.04883 (15)	0.61919 (10)	0.0431 (3)	
C4	0.2058 (2)	1.27426 (19)	0.50197 (15)	0.0524 (4)	
H4	0.1374	1.3442	0.5519	0.063*	
C5	0.2900 (2)	1.3174 (2)	0.40181 (16)	0.0585 (5)	
H5	0.2778	1.4182	0.3837	0.070*	
C6	0.3932 (2)	1.2133 (2)	0.32689 (15)	0.0590 (5)	
H6	0.4492	1.2463	0.2600	0.071*	
C7	0.4145 (2)	1.0629 (2)	0.34927 (14)	0.0526 (4)	
H7	0.4840	0.9934	0.2994	0.063*	
C8	0.32694 (19)	1.01973 (18)	0.45009 (13)	0.0417 (4)	
C9	0.22605 (19)	1.12258 (18)	0.52616 (13)	0.0428 (4)	
C10	0.4119 (2)	0.74040 (19)	0.45014 (13)	0.0455 (4)	
H10A	0.3916	0.6610	0.5026	0.055*	
H10B	0.5254	0.7442	0.4324	0.055*	
C11	0.37785 (18)	0.70334 (17)	0.35053 (12)	0.0389 (4)	
C12	0.23086 (19)	0.75994 (17)	0.33238 (12)	0.0400 (4)	
H12	0.1509	0.8236	0.3818	0.048*	
C13	0.2017 (2)	0.72253 (17)	0.24104 (12)	0.0421 (4)	
C14	0.3202 (2)	0.62518 (19)	0.16856 (13)	0.0506 (4)	
H14	0.3013	0.5981	0.1077	0.061*	
C15	0.4652 (2)	0.5692 (2)	0.18709 (14)	0.0537 (5)	
H15	0.5444	0.5039	0.1383	0.064*	
C16	0.4962 (2)	0.60800 (19)	0.27712 (13)	0.0482 (4)	
H16	0.5959	0.5702	0.2881	0.058*	
C17	0.16366 (18)	0.78669 (18)	0.67598 (12)	0.0404 (4)	
C18	0.18420 (18)	0.78701 (17)	0.77632 (12)	0.0410 (4)	
H18	0.2303	0.8601	0.7927	0.049*	
C19	0.13664 (19)	0.67954 (18)	0.85205 (12)	0.0429 (4)	
C20	0.0629 (2)	0.57380 (19)	0.82858 (14)	0.0498 (4)	
H20	0.0278	0.5028	0.8798	0.060*	
C21	0.0419 (2)	0.5743 (2)	0.72924 (15)	0.0554 (5)	
H21	−0.0072	0.5029	0.7136	0.066*	
C22	0.0925 (2)	0.6792 (2)	0.65240 (14)	0.0524 (4)	
H22	0.0788	0.6777	0.5852	0.063*	
C23	0.2768 (3)	0.0778 (2)	0.11599 (16)	0.0656 (5)	
H23A	0.2826	−0.0043	0.1655	0.098*	
H23B	0.2112	0.1656	0.1540	0.098*	
H23C	0.3835	0.0949	0.0804	0.098*	
C24	0.2345 (3)	0.1831 (3)	−0.07215 (18)	0.0875 (7)	
H24A	0.3493	0.1756	−0.0984	0.131*	
H24B	0.1844	0.2768	−0.0372	0.131*	
H24C	0.1936	0.1767	−0.1309	0.131*	

### Atomic displacement parameters (Å^2^)

**Table:**

	*U*^11^	*U*^22^	*U*^33^	*U*^12^	*U*^13^	*U*^23^
S1	0.0502 (3)	0.0617 (3)	0.0535 (3)	−0.0095 (2)	−0.0182 (2)	−0.0014 (2)
O1	0.0558 (8)	0.0622 (8)	0.0513 (7)	−0.0008 (6)	−0.0261 (6)	−0.0111 (6)
O2	0.0754 (9)	0.0598 (8)	0.0412 (7)	−0.0147 (7)	−0.0218 (6)	0.0059 (6)
O3	0.0678 (8)	0.0618 (8)	0.0617 (8)	−0.0089 (7)	−0.0206 (7)	−0.0126 (6)
N1	0.0410 (7)	0.0417 (8)	0.0385 (7)	−0.0093 (6)	−0.0155 (6)	−0.0009 (6)
C2	0.0398 (8)	0.0451 (9)	0.0378 (8)	−0.0094 (7)	−0.0176 (7)	−0.0025 (7)
N3	0.0457 (8)	0.0430 (8)	0.0430 (7)	−0.0095 (6)	−0.0160 (6)	−0.0011 (6)
C4	0.0572 (11)	0.0447 (10)	0.0618 (11)	−0.0121 (8)	−0.0259 (9)	−0.0007 (8)
C5	0.0646 (12)	0.0502 (11)	0.0734 (13)	−0.0230 (10)	−0.0350 (11)	0.0155 (10)
C6	0.0592 (11)	0.0679 (13)	0.0573 (11)	−0.0285 (10)	−0.0220 (10)	0.0174 (10)
C7	0.0501 (10)	0.0625 (12)	0.0474 (10)	−0.0175 (9)	−0.0145 (8)	0.0022 (9)
C8	0.0422 (9)	0.0468 (9)	0.0429 (9)	−0.0135 (7)	−0.0198 (7)	0.0019 (7)
C9	0.0442 (9)	0.0454 (9)	0.0465 (9)	−0.0134 (7)	−0.0218 (8)	0.0006 (7)
C10	0.0413 (9)	0.0474 (9)	0.0479 (9)	−0.0035 (7)	−0.0168 (8)	−0.0021 (8)
C11	0.0409 (8)	0.0348 (8)	0.0392 (8)	−0.0087 (7)	−0.0098 (7)	0.0028 (7)
C12	0.0398 (8)	0.0372 (8)	0.0406 (9)	−0.0054 (7)	−0.0093 (7)	−0.0046 (7)
C13	0.0478 (9)	0.0380 (9)	0.0420 (9)	−0.0103 (7)	−0.0154 (7)	0.0023 (7)
C14	0.0649 (12)	0.0473 (10)	0.0381 (9)	−0.0087 (9)	−0.0138 (8)	−0.0057 (8)
C15	0.0560 (11)	0.0495 (10)	0.0446 (10)	0.0003 (9)	−0.0052 (8)	−0.0069 (8)
C16	0.0414 (9)	0.0478 (10)	0.0490 (10)	−0.0033 (8)	−0.0086 (8)	0.0003 (8)
C17	0.0382 (8)	0.0424 (9)	0.0413 (9)	−0.0068 (7)	−0.0136 (7)	−0.0012 (7)
C18	0.0394 (8)	0.0409 (9)	0.0427 (9)	−0.0065 (7)	−0.0126 (7)	−0.0035 (7)
C19	0.0416 (9)	0.0419 (9)	0.0412 (9)	−0.0003 (7)	−0.0120 (7)	−0.0013 (7)
C20	0.0529 (10)	0.0400 (9)	0.0506 (10)	−0.0088 (8)	−0.0085 (8)	0.0042 (8)
C21	0.0616 (11)	0.0486 (10)	0.0621 (11)	−0.0227 (9)	−0.0190 (9)	−0.0030 (9)
C22	0.0604 (11)	0.0569 (11)	0.0483 (10)	−0.0207 (9)	−0.0222 (9)	−0.0011 (8)
C23	0.0727 (13)	0.0642 (13)	0.0660 (13)	−0.0122 (10)	−0.0292 (11)	−0.0068 (10)
C24	0.0954 (18)	0.0770 (16)	0.0777 (16)	−0.0072 (14)	−0.0207 (14)	0.0205 (13)

### Geometric parameters (Å, º)

**Table:**

S1—O3	1.5040 (14)	C11—C12	1.383 (2)
S1—C24	1.768 (2)	C11—C16	1.383 (2)
S1—C23	1.7739 (18)	C12—C13	1.388 (2)
O1—C13	1.353 (2)	C12—H12	0.9300
O1—H1	0.857 (9)	C13—C14	1.387 (2)
O2—C19	1.3541 (19)	C14—C15	1.368 (3)
O2—H2	0.848 (9)	C14—H14	0.9300
N1—C2	1.3689 (19)	C15—C16	1.385 (2)
N1—C8	1.384 (2)	C15—H15	0.9300
N1—C10	1.456 (2)	C16—H16	0.9300
C2—N3	1.317 (2)	C17—C22	1.385 (2)
C2—C17	1.471 (2)	C17—C18	1.388 (2)
N3—C9	1.387 (2)	C18—C19	1.382 (2)
C4—C5	1.374 (3)	C18—H18	0.9300
C4—C9	1.391 (2)	C19—C20	1.386 (2)
C4—H4	0.9300	C20—C21	1.375 (2)
C5—C6	1.392 (3)	C20—H20	0.9300
C5—H5	0.9300	C21—C22	1.380 (2)
C6—C7	1.375 (3)	C21—H21	0.9300
C6—H6	0.9300	C22—H22	0.9300
C7—C8	1.390 (2)	C23—H23A	0.9600
C7—H7	0.9300	C23—H23B	0.9600
C8—C9	1.388 (2)	C23—H23C	0.9600
C10—C11	1.512 (2)	C24—H24A	0.9600
C10—H10A	0.9700	C24—H24B	0.9600
C10—H10B	0.9700	C24—H24C	0.9600
			
O3—S1—C24	105.19 (10)	O1—C13—C14	117.93 (14)
O3—S1—C23	106.18 (9)	O1—C13—C12	122.59 (14)
C24—S1—C23	98.83 (11)	C14—C13—C12	119.47 (15)
C13—O1—H1	110.5 (14)	C15—C14—C13	119.66 (15)
C19—O2—H2	111.5 (14)	C15—C14—H14	120.2
C2—N1—C8	106.73 (13)	C13—C14—H14	120.2
C2—N1—C10	128.31 (13)	C14—C15—C16	121.20 (16)
C8—N1—C10	124.60 (13)	C14—C15—H15	119.4
N3—C2—N1	112.33 (14)	C16—C15—H15	119.4
N3—C2—C17	123.64 (14)	C11—C16—C15	119.50 (16)
N1—C2—C17	124.01 (14)	C11—C16—H16	120.3
C2—N3—C9	105.51 (13)	C15—C16—H16	120.3
C5—C4—C9	117.81 (18)	C22—C17—C18	119.58 (15)
C5—C4—H4	121.1	C22—C17—C2	121.67 (14)
C9—C4—H4	121.1	C18—C17—C2	118.67 (14)
C4—C5—C6	121.35 (17)	C19—C18—C17	120.52 (15)
C4—C5—H5	119.3	C19—C18—H18	119.7
C6—C5—H5	119.3	C17—C18—H18	119.7
C7—C6—C5	121.67 (17)	O2—C19—C18	122.55 (15)
C7—C6—H6	119.2	O2—C19—C20	117.87 (15)
C5—C6—H6	119.2	C18—C19—C20	119.58 (15)
C6—C7—C8	116.73 (18)	C21—C20—C19	119.72 (16)
C6—C7—H7	121.6	C21—C20—H20	120.1
C8—C7—H7	121.6	C19—C20—H20	120.1
N1—C8—C9	105.65 (14)	C20—C21—C22	120.98 (16)
N1—C8—C7	132.22 (16)	C20—C21—H21	119.5
C9—C8—C7	122.13 (16)	C22—C21—H21	119.5
N3—C9—C8	109.77 (14)	C21—C22—C17	119.58 (16)
N3—C9—C4	129.92 (16)	C21—C22—H22	120.2
C8—C9—C4	120.29 (16)	C17—C22—H22	120.2
N1—C10—C11	113.71 (13)	S1—C23—H23A	109.5
N1—C10—H10A	108.8	S1—C23—H23B	109.5
C11—C10—H10A	108.8	H23A—C23—H23B	109.5
N1—C10—H10B	108.8	S1—C23—H23C	109.5
C11—C10—H10B	108.8	H23A—C23—H23C	109.5
H10A—C10—H10B	107.7	H23B—C23—H23C	109.5
C12—C11—C16	119.56 (15)	S1—C24—H24A	109.5
C12—C11—C10	121.69 (14)	S1—C24—H24B	109.5
C16—C11—C10	118.74 (14)	H24A—C24—H24B	109.5
C11—C12—C13	120.60 (15)	S1—C24—H24C	109.5
C11—C12—H12	119.7	H24A—C24—H24C	109.5
C13—C12—H12	119.7	H24B—C24—H24C	109.5
			
C8—N1—C2—N3	0.23 (17)	N1—C10—C11—C16	156.07 (14)
C10—N1—C2—N3	173.56 (13)	C16—C11—C12—C13	−0.3 (2)
C8—N1—C2—C17	178.88 (13)	C10—C11—C12—C13	−179.05 (14)
C10—N1—C2—C17	−7.8 (2)	C11—C12—C13—O1	−177.93 (14)
N1—C2—N3—C9	0.35 (17)	C11—C12—C13—C14	1.2 (2)
C17—C2—N3—C9	−178.30 (13)	O1—C13—C14—C15	178.17 (16)
C9—C4—C5—C6	−0.4 (3)	C12—C13—C14—C15	−1.0 (2)
C4—C5—C6—C7	0.6 (3)	C13—C14—C15—C16	0.0 (3)
C5—C6—C7—C8	0.5 (3)	C12—C11—C16—C15	−0.7 (2)
C2—N1—C8—C9	−0.70 (16)	C10—C11—C16—C15	178.00 (15)
C10—N1—C8—C9	−174.35 (12)	C14—C15—C16—C11	1.0 (3)
C2—N1—C8—C7	179.60 (16)	N3—C2—C17—C22	121.37 (18)
C10—N1—C8—C7	6.0 (3)	N1—C2—C17—C22	−57.1 (2)
C6—C7—C8—N1	177.93 (16)	N3—C2—C17—C18	−55.6 (2)
C6—C7—C8—C9	−1.7 (2)	N1—C2—C17—C18	125.91 (16)
C2—N3—C9—C8	−0.81 (17)	C22—C17—C18—C19	1.1 (2)
C2—N3—C9—C4	177.74 (16)	C2—C17—C18—C19	178.10 (14)
N1—C8—C9—N3	0.94 (16)	C17—C18—C19—O2	178.41 (14)
C7—C8—C9—N3	−179.32 (14)	C17—C18—C19—C20	−2.2 (2)
N1—C8—C9—C4	−177.77 (14)	O2—C19—C20—C21	−178.78 (15)
C7—C8—C9—C4	2.0 (2)	C18—C19—C20—C21	1.8 (2)
C5—C4—C9—N3	−179.26 (16)	C19—C20—C21—C22	−0.3 (3)
C5—C4—C9—C8	−0.8 (2)	C20—C21—C22—C17	−0.8 (3)
C2—N1—C10—C11	121.77 (16)	C18—C17—C22—C21	0.4 (2)
C8—N1—C10—C11	−66.00 (19)	C2—C17—C22—C21	−176.51 (16)
N1—C10—C11—C12	−25.2 (2)		

### Hydrogen-bond geometry (Å, º)

Cg1, Cg2 and Cg3 are the centroids of the C4–C9, C11–C16 and C17–C22 rings, respectively.

**Table:**

*D*—H···*A*	*D*—H	H···*A*	*D*···*A*	*D*—H···*A*
O2—H2···O3^i^	0.85 (1)	1.83 (1)	2.6804 (19)	176 (2)
O2—H2···S1^i^	0.85 (1)	2.84 (1)	3.6209 (14)	154 (2)
O1—H1···N3^ii^	0.86 (1)	1.88 (2)	2.7316 (19)	172 (2)
C18—H18···O3^i^	0.93	2.58	3.260 (2)	130
C23—H23*C*···O3^iii^	0.96	2.72	3.643 (3)	162
C10—H10*B*···*Cg*1^iv^	0.97	2.95	3.622 (2)	127
C5—H5···*Cg*2^v^	0.93	2.76	3.624 (2)	156
C23—H23*B*···*Cg*3^vi^	0.96	2.86	3.679 (2)	144

Symmetry codes: (i) *x*, *y*+1, *z*+1; (ii) −*x*, −*y*+2, −*z*+1; (iii) −*x*+1, −*y*, −*z*; (iv) −*x*+1, −*y*+2, −*z*+1; (v) *x*, *y*+1, *z*; (vi) −*x*, −*y*+1, −*z*+1.
